# Supplementary material for: C2CD4B Evokes Oxidative Stress and Vascular Dysfunction via a PI3K/Akt/PKCα–Signaling Pathway
Source: Antioxidants (Basel). 2024 Jan 14;13(1):101. doi: 10.3390/antiox13010101 (PMC10812653; doi:10.3390/antiox13010101)
Supplement: Supplementary file 1 [file antioxidants-13-00101-s001.zip › antioxidants-2771827-supplementary.pdf]

SUPPLEMENTARY DATA

C2CD4B evokes oxidative stress and vascular dysfunction via a PI3K/Akt/PKCα-signaling pathway

Paola Di Pietro [1], Angela Carmelita Abate [1], Valeria Prete [1], Antonio Damato [2], Eleonora Venturini [2], Maria Rosaria Rusciano [1], Carmine Izzo [1], Valeria Visco [1], Michele Ciccarelli [1], Carmine Vecchione [1,2] and Albino Carrizzo [1,2*]

Supplementary results

The ability of C2CD4B to promote PI3K-dependent eNOS uncoupling was further corroborated by additional experiments in which we test the effect of increasing concentrations of β-ME on the ratio between monomer and dimer, as β-ME is reported to influence the dimeric and monomeric form of eNOS [57]. Our result demonstrated that, while in the presence of 0% β-Me oligomers and the monomer form were markedly present, at 1.5% β-Me oligomers were not clearly detectable, with a marked increase of dimer signals. Conversely, 2.5% of β-Me completely abolished the formation of both oligomers and dimeric form of eNOS, resulting in the visualization only of the monomeric form (Supplementary Figure 2).

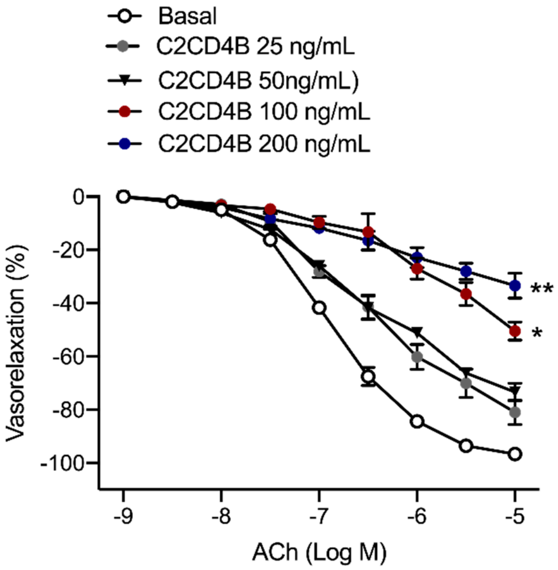

Supplementary Figure S1. Experimental setup for the assessment of the effects of recombinant C2CD4B on mice mesenteric arteries. Acetylcholine (ACh)-evoked vasorelaxation in mice mesenteric arteries exposed to vehicle (basal) or different doses of recombinant C2CD4B protein for 1 hour; (n = 3).

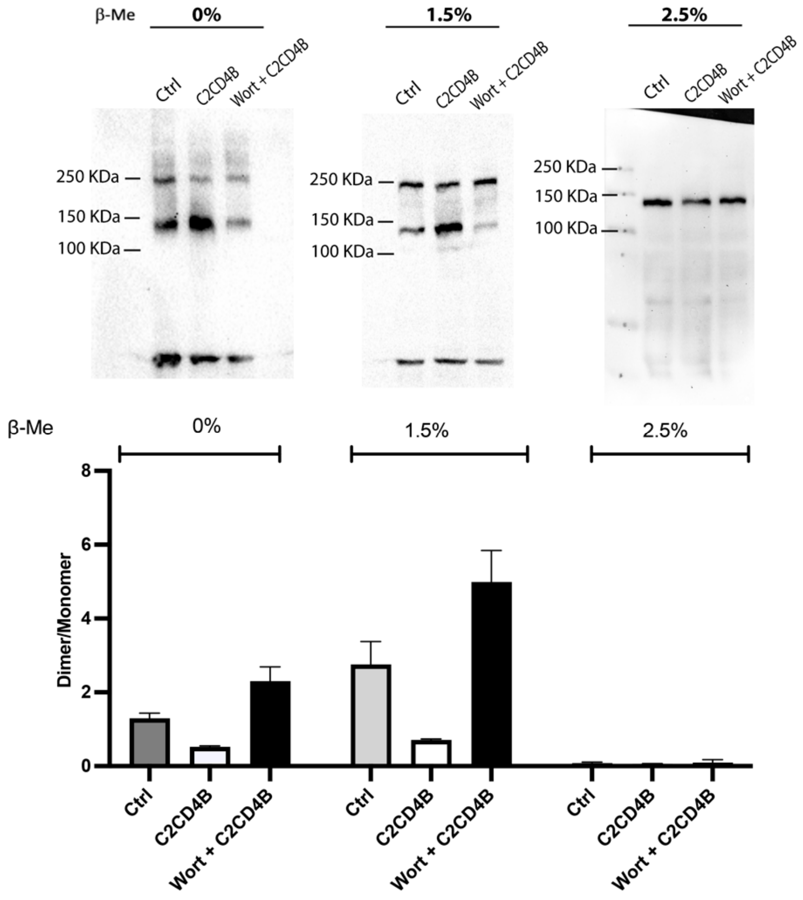

Supplementary Figure S2. Effect of incubation of cell lysates from endothelial cells in the presence or the absence of the reducing agent β-mercaptoethanol (1.5%, 2.5%) on levels of endothelial nitric oxide synthase (eNOS). Representative western blot and densitometric analyses of 3 independent experiments evaluating eNOS dimers and eNOS monomers protein expression in HUVECs treated with vehicle (ctrl), recombinant C2CD4B alone, or pre-treated with wortmannin. Dimer/monomer eNOS were examined by low-temperature SDS-PAGE without (non-reducing) or with (reducing) 2-mercaptoethanol (β-Me, 1.5%, or 2.5%).
